# How Does the Immune System Enter the Brain?

**DOI:** 10.3389/fimmu.2022.805657

**Published:** 2022-02-22

**Authors:** Josephine A. Mapunda, Houyam Tibar, Wafa Regragui, Britta Engelhardt

**Affiliations:** ^1^ Theodor Kocher Institute, University of Bern, Bern, Switzerland; ^2^ Medical School of Rabat, Mohamed 5 University, Rabat, Morocco; ^3^ Hôpital des spécialités de Rabat, Ibn Sina University Hospital of Rabat, Rabat, Morocco

**Keywords:** blood-brain barrier, blood-cerebrospinal fluid barrier, immune cell trafficking, arachnoid barrier, multiple sclerosis

## Abstract

Multiple Sclerosis (MS) is considered the most frequent inflammatory demyelinating disease of the central nervous system (CNS). It occurs with a variable prevalence across the world. A rich armamentarium of disease modifying therapies selectively targeting specific actions of the immune system is available for the treatment of MS. Understanding how and where immune cells are primed, how they access the CNS in MS and how immunomodulatory treatments affect neuroinflammation requires a proper knowledge on the mechanisms regulating immune cell trafficking and the special anatomy of the CNS. The brain barriers divide the CNS into different compartments that differ with respect to their accessibility to cells of the innate and adaptive immune system. In steady state, the blood-brain barrier (BBB) limits immune cell trafficking to activated T cells, which can reach the cerebrospinal fluid (CSF) filled compartments to ensure CNS immune surveillance. In MS immune cells breach a second barrier, the glia limitans to reach the CNS parenchyma. Here we will summarize the role of the endothelial, epithelial and glial brain barriers in regulating immune cell entry into the CNS and which immunomodulatory treatments for MS target the brain barriers. Finally, we will explore current knowledge on genetic and environmental factors that may influence immune cell entry into the CNS during neuroinflammation in Africa.

## Introduction

The human immune system has evolved to protect the body from microbial pathogens and trauma and thus ultimately to ensure host survival in a hostile environment (1). The skin as the outer body surface and the gut and respiratory tracts as the inner body surfaces are the most exposed sites for infection and injury. Their epithelial linings form highly specialized antimicrobial barriers towards the outside and are further fortified by site-specific immune defense mechanisms established by cells of the innate and adaptive immune system [summarized in (2)]. Melanization of the skin has been recognized as an essential component of skin innate immunity with melanocytes and melanin exerting antimicrobial functions [summarized in (3)]. Microbial or traumatic injury elicits a rapid stereotypic activation of tissue-resident innate immune mechanisms that allow for the killing of the microbes and the resolution of the inflammatory response. The innate immune response includes activation of tissue-resident dendritic cells (DCs), which will take up and process the antigens and travel *via* the afferent lymphatic vessels to the tissue-draining lymph nodes leading to activation of T and B lymphocytes and thus the adaptive immune response and immune memory against the specific microbes to provide an accelerated and amplified immune responses in case of a further encounter with the same antigen. During their priming, naïve lymphocytes are imprinted with navigation programs (expression of a combination of adhesion and chemoattractant receptors) that ensure their site-specific homing. In this context, DCs in gut and skin draining lymph nodes have been shown to play an essential role as they process food-derived vitamin A and ultraviolet-induced vitamin D3, respectively, to imprint gut homing and skin homing trafficking programs as well as site-specific effector functions in naïve lymphocytes summarized in (4)]. Skin complexion, sunlight exposure and dietary patterns will thus have a direct impact on immune cell priming. These site-specific effector functions i.e., production of cytokines, killing of infected tissue cells, and antibody production, ensure elimination of the injurious agent and reconstitution of tissue function and also establish a site-specific cellular immune memory with tissue-resident memory T (TRM) cells (5). Immune surveillance of a given tissue thus relies on drainage by lymphatic vessels to transport antigens and antigen-presenting DCs to the draining lymph nodes, as well as on blood vessels to allow for efficient immune cell trafficking to the respective tissues.

The anatomical location of the CNS within the skull and vertebral column provides robust protection from injury from the outside. Unless there is a penetrating injury, pathogens are thus unlikely to directly reach the CNS, unless they have escaped the innate and adaptive immune defense mechanisms at the outer surfaces of the body. However, the CNS resides behind blood-brain barriers that restrict pathogen and immune cell entry from the periphery into the CNS parenchyma and lacks lymphatic vessels. The CNS thus has a unique relationship with the immune system that differs from that of peripheral organs and is referred to as CNS immune privilege. The discovery of CNS immune privilege is based on the observation that foreign tissues, when grafted to peripheral sites like the skin, are readily rejected, but when grafted into the brain parenchyma, they survive for prolonged durations (6). These organs, in which experimentally implanted tissue grafts are incapable of provoking immunity leading to graft rejection, have since then been referred to as “immune privileged organs” (summarized in (7). CNS immune privilege also extends to innate immune responses as neither injection of bacterial products (8), nor experimental induction of cell death within the CNS parenchyma (9, 10) elicits a rapid infiltration of myelomonocytic cells as observed during the stereotypic innate immune response to such stimuli in peripheral organs (11).

Based on these observations, CNS immune privilege was originally thought to be based on “immune ignorance” where lack of lymphatic vessels and the endothelial blood-brain barrier (BBB) would inhibit the afferent and efferent arm of CNS immunity, respectively [summarized in (12)]. However, the observations that tissue grafts when transplanted into the cerebral ventricles were readily rejected (13, 14) and that foreign tissue grafts transplanted into the brain parenchyma of animals that had previously rejected a skin tissue graft of the same donor were readily destroyed (6) questioned this concept. Observations demonstrating that activated circulating T cells can cross the BBB in the absence of neuroinflammation [summarized in (15)] and that tracers injected into the cerebrospinal fluid (CSF) drain into the deep cervical lymph nodes (16) finally provided direct evidence for afferent and efferent connections of the CNS with the immune system and asked for revisiting the concept of CNS immune privilege. Recent advancements in the establishment of reporter mouse models combined with epifluorescence, near-infrared (NIR), and two-photon (2P) intravital microscopy (IVM) have led to the rediscovery of lymphatic vessels within the dura mater and their contribution to CSF drainage into the deep cervical lymph nodes and the proposal of a “glymphatic system” ensuring efficient mixing of CNS interstitial fluid (ISF) with the CSF (summarized by (17). These observations have led to questioning the existence of CNS immune privilege.

We have proposed that CNS immune privilege does exist but requires proper consideration of the special anatomy of the CNS and especially of the localization and function of the different brain barriers, which divide the CNS into compartments that differ with respect to their accessibility to mediators and cells of the innate and adaptive immune system (12). In this concept, the CNS parenchyma is immune privileged, allowing it to prioritize the proper function of neurons over eliciting an immune response, while the CNS ventricular spaces and border compartments (subarachnoid and perivascular spaces) are dedicated to CNS immunity and thus lack full CNS immune privilege.

## The Brain Barriers

Under physiological conditions, the meningeal, endothelial, epithelial, and glial brain barriers maintain CNS homeostasis by protecting the CNS parenchyma from the constantly changing milieu of the bloodstream (Figure).

### The Leptomeningeal Blood-Cerebrospinal Fluid (CSF) Barrier

**Figure 1:**
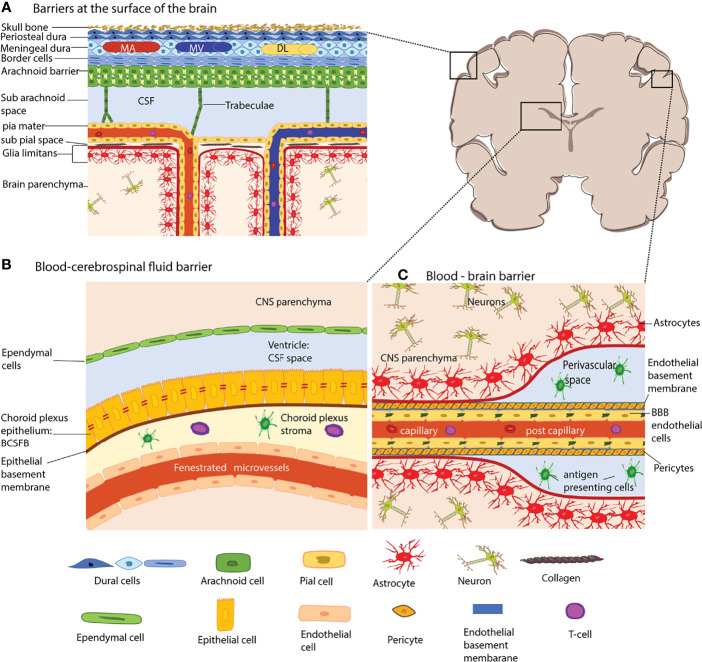
The brain barriers. The schematic coronal brain section depicts the localization of the different brain barriers shown in **(A–C)**. **(A)** Barriers at the surface of the human brain. The meninges are composed of three layers, the dura mater, the arachnoid barrier, and the pia mater. The dura mater is directly connected to the skull bone. In humans, the dura mater is composed of three layers, the periosteal dura, the meningeal dura and the dural border cells. The dura mater has its own network of arteries (MA), veins (MV) and dural lymphatics (DL). The arachnoid barrier is formed by arachnoid fibroblasts which are connected by tight junctions and form a bona fide blood-cerebrospinal fluid barrier (BCSFB) – the arachnoid barrier – between the dura mater and the CSF filled subarachnoid space. Arachnoid trabeculae formed by a collagen core that is ensheathed by arachnoid and pial fibroblasts cross the SAS towards the pia mater and to the leptomeningeal blood vessels. The fibroblasts of the pia mater cover the veins and arteries in the SAS and separate the SAS from the subpial space filled with collagen bundles. The pia mater reflects of the surface where arteries dive into the brain parenchyma and at the same time ensheathes the arteries entering the brain. The glia limitans forms a barrier at all surfaces of the CNS parenchyma, this is the outer surface (*glia limitians superficialis*) and the perivascular surfaces (*glia limitans perivascularis*). **(B)** The blood–CSF barrier of the choroid plexus (ChP). The ChPs are localized in all four ventricles of the brain. The ChP epithelial cells are connected by unique parallel running tight junction stands and establish a BCSFB. The ChP stroma harbors dendritic cells and macrophages and the blood vessels of the ChP are fenestrated. **(C)** The blood–brain barrier (BBB) is formed by highly specialized microvascular endothelial cells connected by complex tight junctions. The endothelial basement membrane harbors a high number of pericytes. At the level of capillaries the endothelial basement membrane and the parenchymal basement membrane of the glia limitans merge. However that the post-capillary venule level they leave a small gap where single antigen-presenting cells can be found. The microvessels are surrounded by the glia limitans, which is composed of the parenchymal basement membrane and astrocyte end-feet. The extravasation of immune cells into the CNS parenchyma occurs at the level of postcapillary venules and thus involves crossing two barriers, the endothelial BBB and after reaching the perivascular space subsequent crossing of the glia limitans. The shapes of the cell types were adapted from Servier Medical Art (http://smart.servier.com/), licensed under a Creative Common Attribution 3.0 Generic License.

The meningeal layers are the dura mater, the arachnoid mater, and the pia mater and cover the entire surface of the brain, and spinal cord (**Figure 1A**) and are mainly composed of fibroblasts (18–20). The dura mater is the outermost layer and is directly attached to the skull. Blood vessels in the dura mater lack a BBB and are thus different from those of the CNS proper (12). Along the superior and transversal sagittal sinuses, the dura mater also harbors lymphatic vessels suggested to drain antigens and immune cells from the CNS (21–23). This would require breaching the arachnoid mater below the dura mater, which establishes a bona fide blood– cerebrospinal fluid barrier (BCSFB) between the dura mater and the CSF filled subarachnoid space (SAS). The arachnoid fibroblasts are connected by tight junctions (24–28) prohibiting free diffusion of solutes and water-soluble molecules across this barrier and also express efflux pumps ensuring transport of toxic metabolites out of the CSF (29).

The arachnoid trabeculae are mainly composed of collagen fibers and fibroblasts that add rigidity to the arachnoid barrier allowing to form a prominent SAS (30). The pia mater is formed by a single layer of flattened fibroblasts covering the surface of the brain and the spinal cord. The cells of the pia mater do not form tight junctions, thus making them permeable to solutes while however still limiting the passage of cellular elements like erythrocytes (31). Additionally, the pia mater sheathes all blood vessels in the SAS and does separate the SAS from the perivascular spaces by reflecting off the surface of the brain (31–33).

### The Glia Limitans

The glia limitans envelops the brain and spinal cord parenchyma’s entire surface and the perivascular spaces. The glia limitans is composed of a parenchymal basement membrane produced by astrocytes and by astrocyte endfeet (12). In the healthy CNS, the polarized expression of the water channel aquaporin 4 (AQP4) in astrocyte endfeet regulates water transport at this barrier. In addition, astrocyte endfeet are joined together by gap junctions that allow for communication between the astrocytes (34). In the healthy CNS, the glia limitans provides a barrier for immune cells scanning the subarachnoid and perivascular spaces and prohibits their uncontrolled entry into the CNS parenchyma (35, 36).

### The Endothelial Blood-Brain Barrier (BBB)

The endothelial BBB forms a barrier between the blood and the CNS. It is established by brain microvascular endothelial cells that are joined together by continuous and complex tight junctions which inhibit free paracellular diffusion of solutes and water-soluble molecules (37, 38) (**Figure 1C**). Combined with the low vesicular activity of BBB endothelial cells that prohibits uncontrolled transcellular diffusion, the BBB establishes a physical barrier for solutes and water-soluble molecules. Expression of specific enzymes, transporters, and efflux pumps make BBB endothelial cells biochemically unique and ensure the transport of nutrients into the CNS and toxic metabolites out of the CNS (38). BBB tight junctions are composed of the transmembrane proteins claudin-5, occludin, and members of the junctional adhesion molecules (JAM). While claudin-5 establishes a diffusion barrier for small molecules (39), occludin regulates calcium movement across the BBB and in addition to TJ stability and barrier function (40). JAM-A, JAM-B and JAM-C have been described in the brain microvascular endothelial cells and have been suggested to play a role in regulating the BBB stability by some but not others (41, 42). Members of the JAM family may however play a role in immune cell migration across the BBB (43, 44). Prerequisites for TJ formation are adherens junctions (AJs), and thus, in addition to their unique TJs, BBB endothelial cells display regular endothelial AJs [summarized in (37)]. VE-cadherin is the main transmembrane protein of the endothelial AJs and keeps neighboring cells attached by homophilic interactions (45). Additional transmembrane proteins localized to BBB cell-to-cell junctions are the platelet endothelial cell adhesion molecule-1 (PECAM-1) that contributes to vascular integrity (46) and CD99 which mediates leukocyte trafficking across the BBB (47).

The unique BBB phenotype in CNS microvascular endothelial cells is not intrinsic to the endothelial cells but relies on the continuous cross-talk with cellular and acellular elements surrounding the CNS microvascular endothelium forming the neurovascular unit (NVU). BBB endothelial cells produce the endothelial basement membrane composed of type IV collagen, α4 and α5 laminins (36). Additionally, a high number of pericytes is embedded in the endothelial basement membrane at the level of capillaries and possibly post-capillary venules, while smooth muscle cells form the mural cell population in arterioles and possibly venules (48). The CNS blood vessels are always separated from the CNS parenchyma proper by the glia limitans. The parenchymal basement membrane, which is secreted by the astrocytes, is with the expression of a1 and a2 laminin molecularly distinct from the endothelial basement membrane (36, 49, 50). At the capillary level, the parenchymal basement membrane fuses with the endothelial basement membrane bringing the astrocyte endfeet in close proximity to capillary pericytes and endothelial cells. At the level of the post-capillary venules, the two basement membranes detach to form a small perivascular space (51).

### The Choroid Plexus and the Blood-Cerebrospinal Fluid Barrier (BCSFB)

The choroid plexus (ChP) extends into all four brain ventricles and is surrounded by epithelial cells that form a blood-cerebrospinal fluid barrier (BCSFB) (52) (**Figure 1B**). The ChP produces CSF and ChP epithelial cells are characterized by the expression of a particular combination of transporters (53). Paracellular diffusion across the ChP BCSFB is prohibited by unique tight junctions composed of claudin-1, -2, -3, and -11, occludin, and JAM-A and the scaffolding proteins ZO-1, -2, -3 (54). The capillaries in the choroid plexus stroma are fenestrated and thus allowing for free diffusion of blood-borne molecules into the ChP stroma. The ChP stroma harbors numerous cells of the innate but also the adaptive immune system (55). Furthermore, on the apical side of the ChP epithelial cells, epiplexus or Kolmer cells perform immune surveillance.

## The Role of the Individual Brain Barriers in Regulating Immune Cell Entry Into the CNS

### Immune Cell Trafficking Across the Endothelial Blood-Brain Barrier (BBB)

CNS immune surveillance has been shown to be ensured by peripherally activated circulating T cells that have the specific ability to cross the BBB to reach perivascular or subarachnoid spaces in the absence of neuroinflammation (12, 56). It should be noted that while immune cells trafficking occurs at the level of CNS post-capillary venules, transport of nutrients occurs at the level of CNS capillaries (57). This allows immune cells to reach perivascular or subarachnoid space, where they can encounter tissue resident antigen-presenting cells (APCs), like border associated macrophages (BAMs). Recognition of their cognate antigen on these CNS border associated APCs leads to local reactivation of T cells and is the prerequisite for subsequent T-cell migration across the glia limitans into the CNS parenchyma (58, 59).

**Figure 2:**
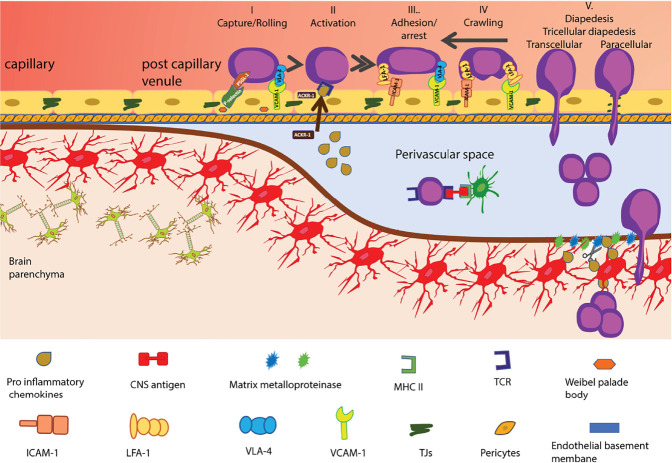
Multi-step T-cell extravasation across the BBB during neuroinflammation. Multi-step T cell extravasation across the BBB occurs at the level of CNS post capillary venules. During inflammation, the rolling of activated T-cells on the BBB endothelial cells is mediated by P-selectin and a4-integrins. After their GPCR-dependent arrest, T cells crawl on the BBB endothelium against the direction of blood flow. High levels of endothelial ICAM-1 and *de novo* expression of ACKR1 that can shuttle CNS chemokines across the BBB promote transcellular diapedesis of T cells while low levels of endothelial ICAM-1 direct T cells mainly to tricellular and bicellular junctions, i.e. paracellular sites of diapedesis. Once T cells have crossed the BBB endothelium they reach the perivascular space. The CNS-antigen-specific T cells may recognize their cognate antigens on perivascular APCs and become reactivated and start to proliferate. The change in local cytokine milieu leads to induction of matrix metalloproteinases -2 and -9 which cleave extracellular matrix receptors on astrocyte endfeet, allowing for T-cell passage across the glia limitans. Once in the CNS parenchyma, T cells induce tissue injury and clinical disease symptoms start to appear. The shapes of the cell types were adapted from Servier Medical Art (http://smart.servier.com/), licensed under a Creative Common Attribution 3.0 Generic License.

Leucocyte extravasation is usually a multi-step process where after an initial tether or capture on the endothelium, selectins and their ligands allow immune cells to roll along the endothelium, reducing their speed and next recognize with their G-protein coupled receptors (GPCRs) chemotactic cues on the endothelium leading to their subsequent integrin-mediated arrest and crawling and finally their diapedesis across endothelial barrier (60). The unique barrier characteristics of the BBB extend to its characteristic immune quiescent phenotype. In contrast to peripheral endothelial cells, BBB endothelial cells lack storage of P-selectin protein in their endothelial Weibel Palade bodies [summarized in (61)] and constitutive expression of the atypical chemokine receptor 1 (ACKR1), which transports chemokines from the abluminal to the luminal surface of endothelial cells (62, 63). Thus, immune cell entry into the CNS is very low and limited to activated T cells that do not depend on these trafficking cues. Indeed, activated CD4 T cells were shown to be able to capture *via* α4-integrins on CNS endothelial VCAM-1 (64) and following LFA-1 dependent adhesive interactions to cross the BBB in the absence of neuroinflammation (65–67) (**Figure 2**).

At onset of neuroinflammation, T cells have been shown to cross venules in the subarachnoid space and crawl within the subarachnoid space, where they can also be washed off with the CSF (58).

During neuroinflammation, *de novo* expression of trafficking molecules like P-selectin and ACKR1 allow for increased immune cell entry into the CNS. The interaction between the P-selectin glycoprotein ligand (PSGL)-1 on T cells and E- and P-selectin on the BBB allows for tethering and rolling of activated CD4 T cells along the luminal side of inflamed spinal cord microvessels (68, 69). Rolling on the BBB allows T cells to interact with chemokines displayed on proteoglycans on the luminal surface of the endothelial cells *via* their specific GPCRs or possibly on ACKR1, leading to an inside-out-activation of integrins mediating the firm arrest of the immune cells on the luminal surface of the inflamed BBB endothelial cells (61). The interaction between the integrins LFA-1 and very late antigen-4 (VLA-4, α4β1 integrin) on the T cells and their endothelial ligands, ICAM-1 and VCAM-1, respectively mediates the firm adhesion of T cells to the BBB (70, 71). After their arrest, the T cells polarize and were observed to crawl over extended distances against the direction of the bloodstream on endothelial ICAM-1 and ICAM-2, obviously to find rare tricellular junctions as sites permissive for diapedesis across the BBB endothelium (58, 66, 67). Under neuroinflammatory conditions, high cell surface levels of endothelial ICAM-1 and *de novo* expression of ACKR-1 were shown to reduce T cell crawling distances and increase transcellular T-cell diapedesis across the BBB (62, 66). Importantly, although the BBB junctions become leaky under neuroinflammatory conditions and allow for uncontrolled diffusion of blood-borne molecules across the BBB, this is not accompanied by increased paracellular T cell diapedesis but rather leads to enhanced transcellular T cell diapedesis across the BBB. This underscores that the mechanisms that regulate the junctional integrity of the BBB are distinct from those regulating the cellular pathway of T cell diapedesis across the BBB.

In contrast to CD4 T cells, the molecular mechanisms involved in the multi-step migration of other immune cell subsets across the BBB are less well understood but are distinct from those of CD4 T cells. Although CD8 T cells also rely on α4-integrins to cross the BBB, they show enhanced dependence on LFA-1 to mediate shear resistant arrest and engage in addition endothelial JAM-B (72–76) which is not required for CD4 T cell diapedesis across the BBB (41). Also, although α4-integrins seem to be involved in the migration of most immune cell subsets across the BBB, the precise molecular mechanisms involved in every step of the multi-step extravasation of B cells (77, 78) or innate immune cells such as neutrophils (79), monocytes (75, 80–82) and dendritic cells (83–86) to cross the BBB, are not yet fully understood.

Additionally, several studies have proposed that other molecules such as the activated leukocyte cell adhesion molecule (ALCAM) (80, 87) and the melanoma cell adhesion molecule (MCAM) (88) as well as the nerve injury-induced protein (ninjurin-1) (89) might play a role in the migration of T-cells across the BBB during EAE and MS (76). Future studies to determine the precise role of these molecules in immune cell trafficking across the BBB still need to be done.

### Immune Cell Trafficking Across the Glia Limitans

In experimental autoimmune encephalomyelitis (EAE), an animal model for multiple sclerosis (MS), clinical symptoms start only upon immune cells crossing the glia limitans and reaching the CNS parenchyma (41, 90). This underscores that immune cell entry into the CNS is fundamentally different from that in peripheral tissues and involves two sequential and differentially regulated steps of crossing an outer brain barrier followed by progression across the glia limitans into the CNS parenchyma proper (91).

Under normal physiological conditions, the glia limitans act as a barrier for migrating immune cells by preventing their entry into the CNS parenchyma (52). During neuroinflammation, when BBB integrity is impaired, reactive astrocytes form tight junctions aiming to prohibit the parenchymal entry of humoral and cellular factors from the bloodstream (92). Nevertheless, it has also been observed that under neuroinflammatory conditions, such as during MS or its animal model EAE, immune cells first form a perivascular cuff around post-capillary venules and then can cross the glia limitans and infiltrate the CNS parenchyma initiating an onset of neurological symptoms (91). This process is mediated by local TNF-induced expression and activation of matrix metalloproteinase (MMP)-2 and MMP-9, which allow for cleavage of a-dystroglycan, an extracellular matrix receptor of astrocyte endfeet, and modulation of chemokines, thus enabling T-cell migration across the perivascular glia limitans into the CNS parenchyma (90, 93). *In vivo* imaging studies have provided ample evidence that T- cells can cross the walls of the leptomeningeal veins to reach the SAS (94). If this allows for their subsequent migration across the glia limitans on the surface of the brain and spinal cord into the CNS parenchyma is still a matter of debate.

### Immune Cell Trafficking Across the Leptomeningeal Arachnoid Barrier

The role of the arachnoid barrier in regulating immune cell entry into the subarachnoid space is not well investigated. A recent study described the downregulation of claudin-11 in arachnoid barrier cells during EAE and MS. In EAE, the authors detected accumulation of T-cell infiltrates specifically in regions of the spinal cord associated with loss of claudin-11 immunostaining of arachnoid barrier fibroblast (95). This establishes a correlation with impairment of arachnoid barrier fibroblast TJs and CNS immune cell infiltration.

Recent studies have furthermore proposed that the dura mater harbors immune cells dedicated for CNS immune surveillance and directly sourced from nearby bone marrow cavities (96–98). Vice versa, it has been suggested that immune cells can readily reach the dural lymphatics from the subarachnoid space (22, 99).

Furthermore, the accumulation of B-cell follicles observed in the subarachnoid space of post mortem brain samples from progressive MS patients has ignited a discussion on the role played by the meninges in MS pathogenesis (100, 101). These meningeal B cell clusters have originally been described in EAE (102) and recent studies have suggested that these B-cells originate from the dura mater (97, 98) and may or may not migrate from the calvaria to the dura mater through specialized vascular channels traversing the inner skull bone. None of these studies has, however, integrated consideration of the arachnoid barrier which establishes a blood-CSF barrier between the dura mater and the subarachnoid space. Thus, it remains to be shown if the arachnoid barrier is a barrier for immune cell passage into the CNS during immunosurveillance and neuroinflammation.

### Immune Cell Trafficking *via* the Choroid Plexus

The ChP has been proposed as an alternative CNS entry site for immune cells reaching the CSF-filled space during immunosurveillance and in neuroinflammation (55). The ChP microvessels do not form a BBB and have a phenotype rather resembling that of peripheral endothelial cells with e.g., constitutive storage of P-selectin in Weibel Palade bodies (103). To reach the CSF, immune cells would need to cross the BCSFB ensheathing the ChP stroma. Adhesion molecules such as ICAM-1 and VCAM-1 are expressed at the luminal surface of ChP epithelial cells, and *in vitro* studies have provided evidence that T cells can cross the monolayers of ChP epithelial cells from the abluminal to the luminal side with a contribution of epithelial ICAM-1 during the final step of diapedesis and release into the CSF space (104).

CSF from healthy individuals or individuals with non-neuroinflammatory disorders harbors tissue memory CD4+ T helper cells and CD8+ T cells (105, 106). It has been proposed that CSF T cells cross fenestrated capillaries of the ChP in a P-selectin-dependent manner to reach the ChP stroma (103). From there, at least Th17 cells expressing the chemokine receptor CCR6 were suggested to cross the BCSFB expressing the CCR6 ligand CCL20 in a CCR6/CCL20-dependent manner (107). Direct evidence for the migration of T cells from the ChP across the BCSFB *in vivo* awaits application of recently developed advanced imaging methodologies of the ChP (108). It has also been proposed that rather than crossing the BBB, immune cells exit the ChP stroma at the base of the ChP where it folds out from the ventricular wall. The BCSFB basement membrane was proposed to be in direct continuation with the parenchymal basement membrane of the glia limitans superficialis (53), allowing immune cells to crawl along the basement membranes reaching the SAS of the brain. Future studies on the precise anatomy of the base of the ChP are necessary to explore this potential immune cell entry route into the CNS.

### Immune Cell Entry Into the CNS in Autoimmune Disease

MS is considered a prototypic organ-specific autoimmune disease targeting the CNS characterized by inflammatory lesions, brain barriers breakdown, demyelination, and axonal damage. The etiology of MS and its pathogenesis is not fully understood, and environmental and genetic factors have been shown to play a vital role in the development of MS. Many MS-associated genetic variants code for molecules related to the proper function of the immune system is consistent with the concept of MS as a T cell-mediated autoimmune disease of the CNS. Further support for a T-cell mediated autoimmunity in MS is derived from its animal model, experimental autoimmune encephalomyelitis (EAE), where neuro-antigen specific autoreactive CD4 T cells infiltrate the CNS and cause CNS pathology resembling that of MS (109).

Histopathologically, active lesions in early MS are characterized by focal white matter demyelination accompanied by perivenular immune cell infiltrates forming a typical perivascular cuff and consisting mainly of CD8 T cells, CD20 B cells, and plasmablasts as well as macrophages (110, 111). Immune cell trafficking to the CNS is thus central to MS pathogenesis and has been recognized as the therapeutic target for the treatment of MS.

## MS Therapies Targeting Immune Cell Trafficking to the CNS

The options for the treatment of relapsing-remitting MS have significantly grown during the last years. These disease-modifying treatments (DMTs) have in common that they target specific actions of the immune system and come with different side effects. Only few DMTs directly target immune cell trafficking to the CNS. Natalizumab is a humanized function blocking monoclonal antibody binding to the α4-integrin subunit of α4β1-(VLA-4) and α4β1−integrin on the immune cell surface. *In vivo* imaging studies in experimental animals have shown that Natalizumab blocks α4- integrin mediated capture on CNS endothelial VCAM-1 in the absence of neuroinflammation (64) as well as sustained adhesion on inflamed BBB endothelium (112) and thus prohibits T cell migration across the BBB (113). This leads to the reduction of CNS inflammatory lesions with BBB breakdown as well as reduced numbers of CD4 and CD8 T cells detected in the CSF of MS patients (114).

The sphingosine phosphate 1 receptors (S1PR) S1PR1, S1PR3, S1PR4, and S1PR5 are expressed on many cell types including lymphocytes and the BBB and have been shown to be involved in the regulation of many biological processes including lymphocyte trafficking and vascular permeability. Four S1PR modulators, namely fingolimod, siponimod, ozanimod and ponesimod) are currently approved for the treatment of MS (summarized in (115). As S1P signaling is required for the egress of CCR7 expressing lymphocytes from lymph nodes, S1PR modulators trap naïve and central memory cells in lymph nodes while CCR7^neg^ effector memory (T_em_) and effector memory recently activated T cells (T _EMRA_) are not affected (116–118). The resulting lymphopenia and change in composition of circulating lymphocytes is thought to reduce immune cell trafficking into the CNS and is considered the main therapeutic effect of the S1PR modulators in MS. At the same time other effects including a direct effect on the BBB remains to be investigated (113).

In addition to their direct effects on immune cell trafficking glucocorticoids have been described to stabilize adherens and tight junctions of the BBB by upregulating expression of VE-cadherin and occludin and claudin-5 in brain endothelial cells (119). Similarly, interferon-beta has been proposed to restore barrier properties of the BBB which will eventually influence immune cell trafficking into the CNS (120).

## Genetic and Environmental Factors Influencing MS in Africa

MS prevalence is increasing worldwide and shows a heterogeneous distribution globally, with the highest prevalence in Europe and North America (121, 122). In Africa, although MS has not been widely studied, epidemiological reports have shown a diverse distribution of the disease with a higher occurrence in North Africa as compared to Sub-Saharan Africa (122, 123). With the unknown etiology of MS and the complex interplay between genetic and environmental factors involved in disease pathogenesis has been proposed to be necessary for MS development. Most epidemiological studies focusing on the impact of genetic and environmental risk factors on MS development were performed in Caucasian populations with a representation of 85-99% of the population, while with a representation of 56% the African population is under represented (121). Very little information is available on the African populations, which have great genotypic and phenotypic variability, but several studies have shown that being a member of the African population is itself a risk factor in developing a severe course of the disease. This was proven throughout many studies based on different parameters of severity evaluation of the disease ranging from disability scores, radiological activity or even atrophy (124–127).

## Genetic Factors

Genome-wide association studies (GWAS) identified many single nucleotide polymorphisms (SNPs) in genes coding for molecules regulating functions of the immune system (128), which is consistent with the concept of MS being a T-cell mediated autoimmune disease targeting the CNS. There is, however, an overrepresentation of immune cells in the transcriptional, epigenetic and pathway analysis datasets used in the GWAS studies to interpret the relevance of SNPs to MS susceptibility, which naturally favors identification of MS risk factors associated with immune cells. Inclusion of CNS datasets in GWAS is just emerging (129). which may allow to discover additional risk factors outside of the immune system. To this end in Caucasian populations, the most vital genetic link to MS has been found in MHC haplotypes, especially those containing HLA-DRB1*15.01, HLA DQB1*06.02, and DQA1*01.02 (130). The few studies in black Africans have revealed a diverse distribution in the *HLA-DRB1 and -DQB1* loci expression. For instance, a study in Morocco showed a positive association between the HLA-DRB1-15 and the genetic predisposition to MS in a Moroccan population of MS patients (131). This gene has been reported to play a role in immunity, a study showed that in HLA-DR1-15 positive patients, Th1 lymphocytes auto-proliferate in an elevated way and leading to the binding and presentation of CNS antigens to T cells (132). African ethnic groups that have a higher distribution of these alleles are protected against parasitic infections like malaria but are at higher risks of developing autoimmune diseases like MS (133–135). Individuals lacking expression of the atypical chemokine receptor 1 (ACKR1), formally referred to as DARC (Duffy blood group antigen receptor for chemokines) are for example resistant to malaria. ACKR1 mediates inflammatory chemokine shuttling across the BBB and enhances transcellular T-cell diapedesis across the BBB during EAE (62, 63). Lack of ACKR1 ameliorates development of EAE and it remains to be shown if individuals lacking functional ACKR1 are protected from MS. Alternatively, also different ACKR1 haplotypes could affect susceptibility to MS. To this end over 900 ACKR1 haplotypes were identified (136). There is in fact evidence that a strong selective pressure for malaria resistance in the Ethiopian population correlates with the development and maintenance of certain *ACKR1* haplotypes (137). A correlation of ACKR1 haplotypes with susceptibility to MS has not yet been investigated.

There is also first studies highlighting polymorphisms in adhesion molecules, e.g. for ICAM4, among African ethnicities (138). It will be interesting to see if polymorphisms in adhesion molecules involved in MS pathogenesis may influence susceptibility to MS in the African population.

Moreover, studies have shown that color tones of the skin influence MS pathogenesis. In two population-based case control studies done in Australia, the researchers assessed the skin phenotype spectrophotometrically by measuring the melanin density of the skin at the upper inner arm and buttock aiming for body sites that are usually not exposed to sunlight. Both studies assessed the association between the skin phenotype and likelihood of developing MS. They concluded that people with a pale skin had a 32.4% increase of developing first demyelinating events. Additionally, low melanin density at the buttock and fair skin were associated with earlier onset of disease. Suggesting that pale-skinned people have a higher risk of developing MS and show earlier MS symptoms as compared to people with black skin (139, 140).

## Ultraviolet Radiation and Vitamin D Levels

The prevalence of MS increases directly proportional to an increase in distance from the equator. Several studies have confirmed the association between lower sun exposure with lower Vitamin D levels and the increased risk of developing MS. Considering its geographical location, African countries experience more sun exposure during the year compared to other continents (141). Although there are no studies assessing the impact of sun exposure on MS development in African countries, the observed low prevalence of MS in African countries might be due to increased sun exposure.

**Figure 3:**
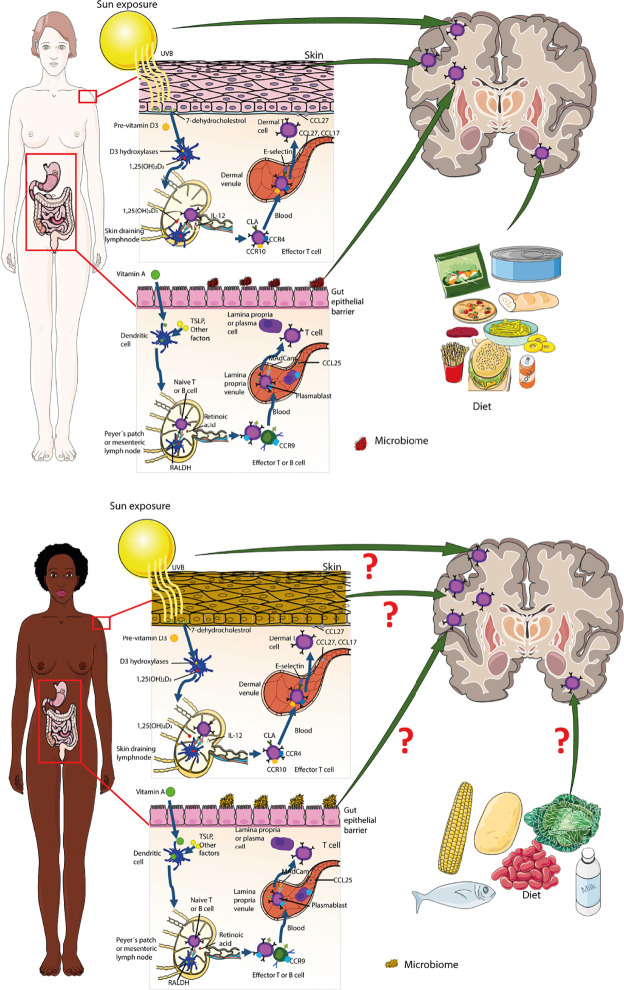
Genetic and environmental factors influencing immune cell entry into the brain. Encounter of microbes takes place at the inner and outer surfaces of the body equipped with special barrier forming epithelia and innate immune cells residing behind these barriers. Priming of T cells in skin and gut-draining lymph nodes imprints their effector function, i.e. expression of trafficking molecules. Pale-skinned people have a higher risk of developing MS as compared to people with black skin. The schematic representation shows imprinting of trafficking properties in T-cells primed in the skin and the gut (adapted from (2), chapter 14). Experimental animal studies have shown that autoagressive T cells primed in skin-draining lymph nodes express CXCR6 and can enter the CNS white and grey matter, while when these T cells are primed in gut-draining lymph odes they express P2rx7 and only infiltrate CNS white matter. How skin color and the gut microbiome of the African population affects T cell priming and their CNS homing properties remains to be shown. The shapes of the cell types were adapted from Servier Medical Art (http://smart.servier.com/), licensed under a Creative Common Attribution 3.0 Generic License.

There is ample epidemiological evidence implicating lack of Vitamin D as a risk for the development of MS. Vitamin D interacts with its specific receptor expressed by all immune cells that influence the transcription rate of Vitamin D responsive genes resulting in strong immunoregulatory effects (142). In addition, in skin-draining lymph nodes DCs metabolize Vitamin D to imprint trafficking and effector programs in naïve T cells (143) (**Figure 3**).

Seasonal differences in MS activity have also been reported. The predicted correlation between sun exposure and increased levels of vitamin D would suggest higher disease activity during low sun exposure seasons such as fall and winter (144). However, recent studies have rather suggested the opposite, namely that disease activity increases during spring and summer (145). Studies from Africa have shown that the majority of the African population have low levels of Vitamin D (146–148). These findings contradict that lower prevalence of MS in Africa is correlated to Vitamin D and rather suggests that immunomodulatory effects of Vitamin D and their potential impact on immune cell trafficking need further investigation (149). Furthermore, many of the reported therapeutic essays of vitamin D supplementation for MS patients still do not prove any efficacy on the EDSS score or annual relapses rate (150). Also studies from us in African MS patients supplemented with high dosage Vitamin D did not now show a significant association between Vitamin D levels and MS status (148).

## Infections

In genetically MS predisposed individuals, studies have shown that microbial infections can act as environmental triggers in inducing or promoting the onset of clinical signs of MS. This has ignited an active debate as to whether infections prevent or precipitate autoimmune diseases [summarized in (151)]. Furthermore, studies conducted in the developed countries have shown that people who were exposed to a higher level of sanitation during childhood had a higher risk of developing MS in adulthood (152) therefore supporting the hypothesis that infections early in live protect rather than induce or accelerate autoimmune diseases like MS.

Further protective evidence of infections in autoimmune diseases was demonstrated by interventional studies where it was reported that individuals treated with anti-helminth drugs showed an increased MS activity (153).

In most African countries, soil-transmitted helminths (STH) affect primarily the people living in rural areas or urban settings with a lack of clean water and poor sanitation (154). STH is still a considerable burden in children aged 5-14 years in Sub Saharan Africa, although a recent study has shown a vast decline in the prevalence of STH in the last decade (155). Even though there are no studies investigating the correlation between STH infections and the risk of developing MS in Africa, we can speculate that exposure to STH during childhood might contribute to the observed low MS prevalence in Africa. Nevertheless, the question remains if we should consider helminths as beneficial commensals or harmful pathogens. Furthermore, if they are beneficial commensals, will deworming the population with anti-helminth drugs cause an increase in autoimmune disorders?

Moreover, there are some viral infections that have been reported to increase the risk of developing MS later in life. A recent study has suggested a causal role of the *Epstein–Barr virus* (EBV) in MS, where MS patients seropositive to EBV had high levels of HLA1-B*07+ genes. They suggested that these HLA-class I molecules present antigens to T lymphocytes and initiate immune response against viruses, and thus supporting the potential role of EBV in MS pathology (156). Prior exposure to EBV has been shown to increase the risk of developing MS in both white and black individuals (157).

Furthermore, a recent meta-analysis has shown a strong association between infection with human herpes viruses (HHV) and MS, suggesting that infection with HHS increases the risk of developing MS although the precise mechanisms remain unclear (158). In addition, few studies have shown a MS protective role of prior *Cytomegalovirus (CMV)* infection (132).

## Gut Microbiota

The gut microbiota plays a vital role in maintaining the host’s homeostasis and preventing inflammatory diseases. Diet is considered as the major driving factor in shaping the gut microbiota across the lifetime (159). Mice raised in the germ-free environment are protected from developing clinical EAE (160). These mice developed EAE only when they were exposed to feces from mice that were colonized with gut microbiota, and the subsequent disease was observed to be very mild, suggesting that the gut microbiota participates in the activation of adaptive immune cells (161). This has been further supported by the observation that transplantation of MS twin-derived microbiota to a transgenic mouse model of spontaneous brain autoimmunity induced a significantly higher incidence of disease when compared to transplantation of the healthy twin -derived microbiota (162). These findings provide evidence for pathogenic microbial components in human MS. Considering the geographical and cultural differences between Europe, America, Asia, Australia and Africa, there is a diverse difference in the gut microbiota, which might impact on MS prevalence (51). Studies comparing protective and pathogenic microbial components in MS in different continents will thus be of fundamental importance to understand if the low prevalence of MS in Africa is also due to a specific gut microbiome affecting the priming and trafficking of immune cell subsets.

## Lifestyle Risk Factors

Both active and passive tobacco smoking has been highly associated with MS onset with a clear dose-dependent relationship. The prevalence rate of tobacco smoking in Africa is low as compared to the Americas and Eastern Mediterranean. However, it is currently increasing at a very high speed when compared to other parts of the world (163). In 2010, the Lancet survey published that Mozambique has seen a 220% growth in cigarette consumption over the past 16 years (164). The increase in the number of smokers is yet to determine if it will increase the prevalence of MS in Africa in the coming years.

Lately, there has been much discussion regarding the contribution of dietary intake to MS incidence and severity. As we know, the diet has a significant influence on the gut microbiome, leading to altered immune function. High salt diet food has been described to promote CD4 T-cell differentiation to Th17 cells, thus leading to earlier disease onset with severe clinical manifestations (165). Furthermore, a high-fat diet has been associated with the development of obesity which puts an individual at a high risk of developing MS (166). The available statistics show increasing trends of body mass index and obesity in Africa (167) which heralds an increase in the MS incidence in the coming years.

## Conclusions

CNS autoimmunity is suggested to be either triggered by molecular mimicry where the adaptive immune response is raised against microbial antigens resembling those of the host or by inflammatory cytokine induced bystander activation, where APCs upregulate, co-stimulatory molecules leading to loss of self-tolerance. The initial activation of these autoaggressive immune cells most likely takes place at outer and inner body surfaces, aka the skin and mucosal surfaces, respectively (**Figure 3**). This has relevance to their CNS trafficking properties. In an EAE model autoaggressive T cells primed in skin draining lymph nodes were shown to infiltrate in addition to the CNS white matter also CNS grey matter using CXCR6 (168). In contrast, autoaggressive T cells primed in gut draining lymph nodes solely infiltrated CNS white matter. Thus, the site of immune cell priming will have a significant impact on T cell effector functions that may not be adequately described with the current immune cell classifications. How skin color and the gut microbiome of the African population affects T cell priming and their CNS homing properties remains to be shown. There is thus an unmet need to compare the specific characteristics of the barrier associated lymphoid tissues in the African population and their impact on immune cell priming during infections to understand the molecular underpinnings of the lower prevalence of MS in Africa and to prevent a future increase of MS in Africa.

## Author Contributions

JM wrote the first draft and compiled all figures. HT and WG wrote part of the document. BE designed the overall layout and edited the entire document. All authors contributed to the article and approved the submitted version.

## Funding

We acknowledge funding of our research by the Swiss National Science Foundation grants CRSII3_154483, 310030E_189312 and 310030_189080 and the Fidelity Bermuda Foundation to BE.

## Conflict of Interest

The authors declare that the research was conducted in the absence of any commercial or financial relationships that could be construed as a potential conflict of interest.

## Publisher’s Note

All claims expressed in this article are solely those of the authors and do not necessarily represent those of their affiliated organizations, or those of the publisher, the editors and the reviewers. Any product that may be evaluated in this article, or claim that may be made by its manufacturer, is not guaranteed or endorsed by the publisher.

## Abbreviations

aEAE, Active EAE; 2P-IVM, Two-photon intravital microscopy; AJs, Adherens junctions; APC, Antigen presenting cell; AQP4, Aquaporin 4; BBB, Blood-brain barrier; BCSFB, Blood-cerebrospinal-fluid barrier; ChP, Choroid plexus; CLDN, Claudin; CNS, Central nervous system; CSF, Cerebrospinal fluid; E-cadherin, Epithelial cadherin; ICAM-1, Intercellular adhesion molecule 1; JAMs, Junctional adhesion molecules; LFA-1, Lymphocyte function-associated antigen 1; NVU, Neurovascular unit; PECAM-1, Platelet endothelial cell adhesion molecule 1; PSGL, P-selectin glycoprotein ligand; STH, soil-transmitted helminths; TJs, Tight junctions; VCAM-1, Vascular cell adhesion molecule 1; VE-cadherin, Vascular endothelial cadherin; ZO, Zona occludens.
